# Treatment outcomes of patients with *Cutibacterium acnes*-positive cultures during total joint replacement revision surgery: a minimum 2-year follow-up

**DOI:** 10.1007/s00402-022-04489-z

**Published:** 2022-06-27

**Authors:** A. Hoch, Y. Fritz, D. Dimitriou, D. A. Bossard, S. F. Fucentese, K. Wieser, Y. Achermann, P. O. Zingg

**Affiliations:** 1 Orthopaedic Department, Balgrist University Hospital, University of Zurich, Forchstrasse 340, 8008 Zurich, Switzerland; 2 Division of Infectious Diseases and Hospital Epidemiology, University Hospital Zurich, University of Zurich, Rämistrasse 100, 8091 Zurich, Switzerland; 3 Internal Medicine, Hospital Zollikerberg, Trichtenhauserstrasse 20, 8125 Zollikerberg, Switzerland

**Keywords:** *C. acnes*, Periprosthetic joint infections, Revision, Outcomes, Total joint replacement

## Abstract

**Introduction:**

Periprosthetic joint infection (PJI) is a devastating complication following total joint replacement (TJR). *Cutibacterium acnes* (*C. acnes*) is a low virulent skin commensal, commonly found during TJR revision surgery for “aseptic” causes. The purpose of the present study was to report the treatment outcomes of patients with *C. acnes* contamination or infection in the presence of a TJR treated with a revision surgery ± implant exchange ± prolonged (≥ 8 weeks) postoperative antibiotics.

**Methods:**

Medical records of patients with at least one positive *C. acnes* culture in intraoperative tissue samples or sonication fluid from a TJR revision surgery between January 2005 and December 2014 were retrospectively evaluated. The primary endpoint was infection eradication according to Delphi criteria. The diagnostic accuracy of preoperative TJR aspiration regarding the diagnosis of *C. acnes* PJI was also investigated.

**Results:**

A total of 52 TJR (28 shoulders, 17 hips, 7 knees) in 52 patients (35 males, 17 females) with an average age of 63 ± 11 (33–86) years were included. At an average follow-up of 67 ± 33 (24–127) months, the infection eradication of *C. acnes* PJI was 97% regardless of the surgical treatment or administration of prolonged postoperative antibiotics. The incidence of unsuspected *C. acnes* PJI was 28.8%. The sensitivity and specificity of preoperative joint aspiration in detecting *C. acnes* PJI were 59% and 88%, whereas the PPV and NNV were 83% and 67%, respectively.

**Conclusion:**

Infection eradication of *C. acnes* PJI was very high at a minimum follow-up of 24 months, suggesting that *C. acnes* PJI could be adequately treated with a combination of revision surgery and prolonged postoperative antibiotics. The preoperative diagnosis of *C. acnes* PJI might be challenging with more than one-quarter of patients presenting without suspicion of *C. acnes* PJI. The appropriate treatment of patients with a single positive culture remains still unclear. A negative TJR aspiration should not rule out a *C. acnes* PJI, especially in the presence of clinical correlates of infection.

**Level of evidence:**

Retrospective case–control study, Level III.

**IRB approval:**

Kantonale Ethikkommission Zürich, BASEC Nr.:2017-00567.

## Introduction

Periprosthetic joint infection (PJI) is a devastating complication following total joint replacement (TJR), as it could jeopardize the outcomes of the procedure, impair patients’ quality of life, and increase morbidity and mortality [1, 2]. The infection can be acquired during implantation or throughout the lifespan of the implant due to hematogenous seeding [3]. As the incidence of TJR increases in the aging population [4], the number of PJIs is expected to rise accordingly, generating a high financial burden to the health care system [5, 6]. The PJI rate varies for different TJR with a reported 1% for primary shoulder arthroplasty [7, 8], 0.3–1.4% for primary hip arthroplasty [7, 9], and 0.5–1.5% for primary knee arthroplasty [10, 11].

*Cutibacterium acnes* (*C. acnes*) is a gram-positive, facultative anaerobic rod and part of the commensal of the skin, colonizing pilous follicles and sebaceous glands mostly in the shoulder and axilla [12]. While low virulent, it might display virulence factors triggering an inflammatory response and promoting bacterial adhesion by forming a biofilm, which occurs promptly in the presence of an orthopedic implant [13]. Although the rate of *C. acnes* PJI is estimated at 0.9–1.9% [13], affecting mostly shoulder but also hip and knee TJR, the diagnosis might be difficult due to the possible lack of clinical symptoms and the fact that the Musculoskeletal Infection Society (MSIS) criteria for PJI [14] might not apply to *C. acnes* PJI. To increase the detection rate of this slow-growing pathogen, a prolonged incubation time was proposed [13, 15]. Going along with this strategy, the probability of *C. acnes* cultivation in deep tissue samples or synovial fluid, as a normal commensal bacterium of the skin, might increase. Furthermore, the incidence of unexpected *C. acnes*-positive cultures during revision shoulder arthroplasty was reported to range from 11.5 to 21.5% [16, 17], with the international consensus meeting on musculoskeletal infection stating that the relevance of unexpected positive cultures in the setting of shoulder arthroplasty is still unknown [18].

Up to date, only a limited number of studies reported the clinical outcomes following treatment of patients with *C. acnes*-positive tissue cultures during TJR revision surgery. Therefore, the purpose of the present study was to report the treatment outcomes (infection eradication, relapse, or reinfection) of patients with *C. acnes* contamination or infection in the presence of TJR treated with a revision surgery ± implant exchange ± prolonged (≥ 8 weeks) postoperative antibiotics. The diagnostic accuracy of preoperative TJR aspiration regarding the diagnosis of *C. acnes* PJI was also investigated.

## Methods

### Study design, inclusion, and exclusion criteria

**Fig. 1:**
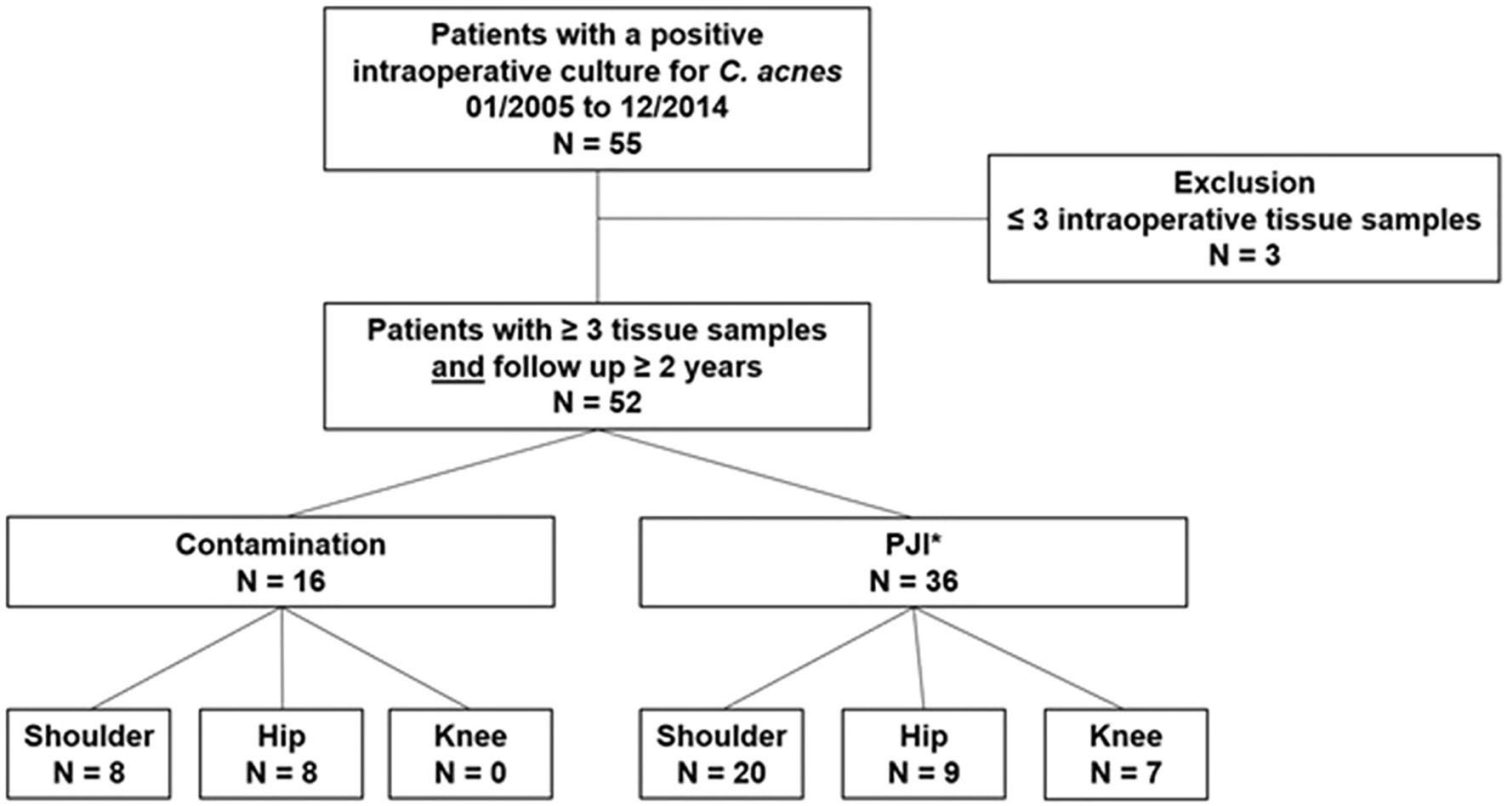
Flowchart of patient selection demonstrating the inclusion, exclusion criteria, and patient classification. *PJI: periprosthetic joint infection was defined according to Asseray et al. [20] criteria as ≥ 2 positive tissue cultures or 1 positive tissue culture and presence of ≥ 2 of the following: (i) history of ≥ 2 local surgeries, (ii) local signs of infection, (iii) intraoperative abnormalities, (iv) increased inflammatory markers, otherwise a single positive culture was considered contamination

The present study was approved by the institutional review board and conducted entirely at the authors’ institution. The medical records of all patients with at least one positive *C. acnes* specimen in intraoperative tissue samples or sonication fluid from a TJR revision surgery between January 2005 and December 2014 were retrospectively evaluated. The inclusion criteria were adult patients, who underwent a TJR revision surgery without antibiotics administration within 2 weeks before surgery, had a positive *C. acnes* tissue sample from at least three intraoperative samples taken, and had a minimum 2-year follow-up after revision surgery at the time of the data collection. Patients who received antibiotics within 2 weeks before surgery, with less than three intraoperative samples or less than 2-year follow-up were excluded from the study (Fig. 1).

### TJR aspiration

Preoperative aspiration of the shoulder, hip, and knee TJR was performed only by clinical suspicion of PJI, under image intensifier guidance following sterile preparation of the skin by experienced radiologists. For sterilization, the skin was rubbed three times with Betaseptic^®^ (Mundipharma, Vienna, Austria) skin disinfectant. If no fluid could be aspirated at the first attempt, the joint was infiltrated with Ringer-lactate and a second attempt was performed. Aspiration fluid was stored in sterile containers and transported to the laboratory for further analysis. The material was then transferred to an aerobic and anaerobic cultivation dish.

### Intraoperative tissue sample processing

The number and location (capsule, synovium, fibrous tissue) of intraoperative cultures varied based on surgeon preference. Acquisition and handling of the tissue specimens were performed under laminar flow. Aerobic and anaerobic cultures were performed for both intraoperative tissue specimens as well as the sonication fluid and were held for 21 days per institutional laboratory protocol for *C. acnes* Microorganisms were identified by standard microbiologic procedures including biochemical characterization with the analytical profile index system (BioMerieux, Nuertingen, Germany). Antibiotic susceptibility testing was performed by disk diffusion or dilution methods according to the clinical and laboratory standards institute guidelines [19]. In addition, a Gram stain was done from all intraoperative tissue specimens as well as sonication fluid on the day of surgery.

### TJR revision surgery and postoperative antibiotic treatment

TJR revision surgery was performed due to pain or prothesis-related complications (e.g., aseptic loosening, stiffness, polyethylene wear, and instability) ± presence of clinical infection signs ± increased inflammatory markers. Patients without preoperative suspicion of PJI were treated with revision surgery and implant retention (IR) or one-stage exchange arthroplasty. In these patients, a PJI was, therefore, diagnosed from positive intraoperative cultures. A standard preoperative antibiotic prophylaxis with cefuroxime was administered at least 30 min before incision in all cases. In patients with a suspected PJI, revision surgery with IR was performed if the symptoms occurred less than 4 weeks from the initial surgery or the onset of symptoms in cases of assumed acute hematogenic infection, otherwise, a one- or two-stage revision was performed. A multidisciplinary team composed of the orthopedic surgeon working closely with an infectious disease and internal medicine specialists decided about the need and duration of postoperative antibiotic administration in a case-based manner, according to clinical infections signs and inflammatory markers.

### Data analysis and primary outcomes

The database of the institute of medical microbiology was used to obtain microbiological data. The patient’s medical history and demographic data were collected using the hospital information system of the department of orthopedics. Patient demographics, date of primary implantation, number of revisions until infection, date of revision surgery, number of taken and positive samples, growth of *C. acnes* ± other pathogens, susceptibility testing, surgical and antibiotic treatment were reported. Asseray et al. [20] criteria were used to differentiate between *C. acnes* PJI from contamination in positive tissue cultures. In summary, *C. acnes* PJI was defined as ≥ 2 positive tissue cultures or 1 positive tissue culture and the presence of ≥ 2 of the following: (1) history of ≥ 2 local surgeries, (2) local signs of infection, (3) intraoperative abnormalities (i.e., loosening), (4) increased inflammatory markers, otherwise, a single positive culture was considered contamination.

The primary endpoint of the present study was infection eradication according to Delphi consensus criteria [21], characterized by: (1) a healed wound without fistula, drainage, pain; (2) no subsequent surgical intervention for infection following revision surgery; or (3) no occurrence of PJI-related mortality, at a minimum 2-year follow-up, as most PJI acquired during revision surgery would have become apparent within that period [22]. Relapse was defined as positive *C. acnes* cultures in two or more diagnostic samples. Reinfection was defined as a new infection with another pathogen.

### Statistical analysis

Descriptive statistics used standard deviation and range to describe all the continuous variables, whereas frequencies and percentages were used to present the discrete variables. The sensitivity, specificity, positive predictive value (PPV), and negative predictive value (NPV) of the preoperative TJR aspiration in detecting *C. acnes* PJI was also calculated.

## Results

### Study population

A total of 55 patients (36 males and 19 females) with at least one positive intraoperative sample of *C. acnes* in the presence of TJR were identified. Three patients were excluded due to insufficient intraoperative samples were obtained (< 3) resulting in 52 patients (Fig. 1). Twenty-eight patients (54%) had a shoulder, 17 (33%) a hip, and 7 (13%) a knee TJR (Table 1). Thirty-six (69%) patients with at least two positive *C. acnes* tissue cultures met the criteria for PJI, whereas the remaining 16 (31%) were considered as contamination (Table 1). The average follow-up after revision surgery was 67.2 (24–127) months.

**Table 1:** Patient characteristics

Characteristics	PJI (*n* = 36)	Contamination (*n* = 16)
Age, mean ± SD (range), years	63 ± 11 (33, 86)	61 ± 10 (38, 83)
Male gender, % (*n*)	69 (25)	63 (10)
TJR		
Shoulder, % (*n*)	56 (20)	50 (8)
Hip, % (*n*)	25 (9)	50 (8)
Knee, % (*n*)	19 (7)	–
Time to revision, mean ± SD (range), months	73 ± 74 (1, 313)	91 ± 101 (5, 286)
Previous surgeries before revision, mean ± SD (range), *n*	2.4 ± 1.7 (1, 7)	3.6 ± 1.9 (1, 8)
Single positive culture, % (*n*)	22.2 (8/36)	100 (16/16)
Positive TJR aspiration, % (*n*)	42 (10/24)	22 (2/9)
Suspected infection preoperative, % (*n*)	72.2 (26/36)	18.8 (3/16)
Intraoperative taken tissue samples, mean ± SD (range), *n*	7.1 ± 2.5 (3, 13)	5.3 ± 1.4 (3, 8)
Growth of additional bacteria (other than *C. acnes*), % (*n*)	31 (11)	13 (2)

*PJI* periprosthetic joint infection, *SD* standard deviation, *TJR* total joint replacement

### Preoperative suspected *C. acnes* PJI

A PJI was preoperatively suspected in 26/36 patients (72.2%) with a later confirmed PJI and in 3/16 patients (18.8%), classified as contamination (Table 1), either due to positive preoperative TJR aspiration in 12 cases (41%) or other abnormalities in the workup in terms of increased inflammatory markers in 8 cases (28%) or clinical signs of infection in 9 cases (31%). In all these cases, the treatment intended to eradicate the suspected PJI, and therefore, all these patients received prolonged postoperative antibiotics. The incidence of unsuspected *C. acnes* PJI was 28.8%. In these patients, the indication for revision surgery were “aseptic reasons” with intraoperative probes obtained as a standard procedure during revision surgery.

### Treatment outcomes of C. acnes PJI

In patients with a confirmed *C. acnes* PJI, revision surgery with IR was performed in 6/36 (17%), a one-staged revision in 12/36 (33%), whereas a two-staged revision in 18/36 (50%) of the patients (Table 2). Prolonged antibiotic treatment was administered in 33/36 (91.6%) of the patients. At a minimum follow-up of 24 months, infection eradication of *C. acnes* PJI was 97% (35/36), whereas the relapse rate was 3% (1/36). No reinfection was observed. In the one case of *C. acnes* relapse, the infected total hip arthroplasty (THA) was exchanged in a two-staged procedure. An antibiotic treatment regimen was applied for 8 weeks (5 days intravenous, 51 days oral). The relapse was diagnosed from intraoperatively obtained samples during reimplantation of the prosthesis, 13 weeks following revision surgery. In another case, a symptomatic PJI with *C. acnes* was diagnosed 6 years following revision surgery for a periprosthetic fracture without preoperative suspicion of a PJI. Therefore, the revision surgery was not performed to eradicate a possible PJI (neither thorough surgical debridement nor a complete exchange of all prosthetic components was performed). As a result, this case was spared for the analysis of the relapse rate in the PJI group. Furthermore, 6 years seems to be a long interval even for a low-grade infection with *C. acnes* to become symptomatic, which could imply either a treatment failure or reinfection with another *C. acnes* strain.

**Table 2:** Surgical treatment and antibiotic regimens

Characteristics	PJI (*n* = 36)	Contamination (*n* = 16)
Surgical treatment		
IR, % (*n*)	17 (6)	25 (4)
One-staged revision, % (*n*)	33 (12)	56 (9)
Two-staged revision, % (*n*)	50 (18)	18. 8 (3)
Time to reimplantation^a^, mean ± SD (range), months	4.8 ± 5 (1, 20)	3.6 ± 1.1 (2, 5)
*Intravenous antibiotics (other than perioperative prophylaxis)*		
Patients, % (*n*)	81 (29)	75 (12)
Duration, mean ± SD (range), days	10.8 ± 6.3 (2, 30)	8.6 ± 5.1 (3, 14)
Regimen, % (*n*)		
Cefuroxime	31 (9/29)	50 (6/12)
Amoxicillin/clavulanic acid	17 (5/29)	–
Vancomycin	31 (9/29)	8 (1/12)
Other	21 (6/29)	42 (5/12)
*Prolonged oral antibiotics (*≥ *8 weeks)*		
Patients, % (*n*)	91.6 (33/36)	62.5 (10/16)
Duration, mean ± SD (range), weeks	12.6 ± 11.4 (4, 52)	9.3 ± 3.8 (6, 12)
Regimen, % (*n*)		
Ciprofloxacin ± rifampicin	30 (10/33)^b^	60 (6/10)
Clindamycin ± rifampicin	30 (10/33)^c^	10 (1/10)
Other	40 (13/33)	30 (3/10)
Follow-up, mean ± SD (range), months	63 ± 31 (24, 127)	73 ± 36 (30, 127)

*IR* implant retention, *PJI* periprosthetic joint infection, *SD* standard deviation, *TJR* total joint replacement

^a^Time to prosthesis reimplantation in patients who underwent a two-stage revision

^b^9 patients had rifampicin along with ciprofloxacin

^c^4 patients had rifampicin along with clindamycin

### Patients with a single positive culture

A total of 24/52 patients (46%) demonstrated a single *C. acnes*-positive tissue culture, with 16 patients demonstrating no local signs of infection, no intraoperative abnormalities, and no increased inflammatory markers (Asseray et al. [20] criteria), and therefore, were classified as contamination (Tables 1, 2). In these patients, revision surgery and IR was performed in 4/16 (25%), a one-staged revision in 9/16 (56%), and a two-staged revision in 3/16 (18.8%) due to preoperative suspicion of PJI (Table 2). In patients with *C. acnes* contamination, no patient had to be revised due to a PJI, irrespective of the treatment regimen.

### Diagnostic accuracy of preoperative TJR aspiration in detecting C. acnes PJI

Of the 33/52 (63.4%) preoperatively obtained TJR aspirations, 12 (36%) were positive for *C. acnes*. In 10/12 (83%) positive aspirations, the presence of a PJI was confirmed intraoperatively (Table 1). Of the 21 negative aspirations, a PJI was confirmed intraoperatively in 7 (33%) cases. In 24/36 (67%) patients with a confirmed PJI, a preoperative TJR aspiration was performed, with only 42% (10/24) of those being positive. The sensitivity and specificity of preoperative TJR aspiration in detecting *C. acnes* PJI were 59% and 88%, while the PPV and NNV were 83% and 67%, respectively.

## Discussion

*Cutibacterium acnes* PJI might be difficult to diagnose due to the possible lack of clinical symptoms and the fact that the MSIS criteria for PJI might not apply to *C. acnes* PJI. As a result, a prolonged incubation time was proposed, increasing the probability of *C. acnes* false-positive intraoperative culture (contamination). The purpose of the present study was to report the treatment outcomes of patients with *C. acnes* contamination or infection based on intraoperative tissue samples during TJR revision surgery. The results of the present study demonstrated that the infection eradication of *C. acnes* PJI was very high regardless of the surgical treatment or administration of prolonged postoperative antibiotics**.** The diagnosis of *C. acnes* PJI might be challenging with more than one-quarter of patients presenting without suspicion of *C. acnes* PJI. The appropriate treatment of patients with a single positive culture remains still unclear. Nevertheless, the relapse rate in these patients is relatively low regardless of the treatment regimen (prolonged antibiotic or simple surveillance). A negative TJR aspiration should not rule out a *C. acnes* PJI, especially in the presence of clinical correlates of infection.

The relapse rate of *C. acnes* PJI following revision surgery and antibiotic treatment is reportedly low. Jacobs et al. [23] in a retrospective study of 60 patients with *C. acnes* PJI and a minimum 1-year follow-up, treated with revision surgery and IR, one-stage or two-staged revision based on the duration of symptoms, and a 3-month postoperative antimicrobial treatment (majority with clindamycin) ± rifampicin, reported a treatment success rate of 93%. Similarly, Zeller et al. [24] reported a 92% infection eradication in 48 shoulders, knee, and shoulder PJI at a 2-year follow-up. In the present study, with a minimum follow-up of 24 months, infection eradication of *C. acnes* PJI following revision surgery ± implant exchange ± prolonged postoperative antibiotics (± rifampicin) was 97% (35/36), regardless of the surgical treatment or administration of prolonged postoperative antibiotics. Regarding the use of rifampicin in the antibiotic regiment, a recent multicenter study reported not superior outcomes in the treatment of *C. acnes* PJI compared to antibiotic regiments without rifampicin [25]. Compared with other organisms, the treatment outcomes of *C. acnes* PJI are favorable. Xu et al. [26] in a retrospective study of 828 PJIs (majority with *staphylococci* or *streptococci* species) reported an overall treatment success of the surgery and antimicrobial therapy of 71.2% (49.4% for IR and 83.0% for two-stage revision) for total knee arthroplasty (TKA) and 68.1% (53.8% for IR and 78.8% for two-stage revision) for THA. These data suggest that *C. acnes* PJI could be adequately treated with a combination of revision surgery and prolonged postoperative antimicrobial treatment with favorable outcomes compared with other organisms. However, it should be noted that, although the well-established Delphi criteria were used to define infection eradication, it might be possible that some patients had a persistent *C. acnes* PJI but due to the mild symptoms, a TJR aspiration or revision surgery was not performed. This is especially true in *C. acnes* PJI of the shoulder, where pain or stiffness might be the only symptoms, with other signs of infection being absent [27]. Nevertheless, a TJR aspiration or biopsy to confirm infection eradication in relatively asymptomatic patients is not indicated.

The incidence of unsuspected PJI following revision for presumed aseptic reasons might be relatively high. Jacobs et al. [28] in a retrospective study of 679, who underwent a revision for presumed aseptic causes following TKA and THA, 7.9% and 12.1%, respectively, transpired to have a PJI (defined as ≥ 2 positive cultures with the same organism), mostly with *C. acnes* or *S. epidermidis*. Similarly, several studies report an incidence of unsuspected PJI between 2 and 13% [29–31]. In the present study, the incidence of unsuspected PJI with *C. acnes*, despite preoperative TJR aspiration in 67% of the patients with a later confirmed PJI, was 28.8%. This further underlines the difficulties in the clinical setting when dealing with a low virulent pathogen such as *C. acnes*. Nevertheless, the relapse rate in patients with unexpected PJI was 0% in the present study.

The clinical significance of a single *C. acnes*-positive intraoperative culture during revision surgery remains unclear. As Lavegre et al. [32] using Asseray et al. [20] criteria reported, a probable infection should be based on the context of associated bacteriological and clinical findings, whereas contamination is a diagnosis of exclusion based on one positive sample without clinical correlates. The appropriate treatment in cases of probable *C. acnes* infection or contamination is also unclear and should be discussed in a multidisciplinary setting considering patient history, clinical signs, inflammatory markers, and intraoperative findings and not hasten to a decision based on a single bacteriological result [33]. Although in cases of contamination the situation might appear straightforward with simple patient surveillance suggested [33], in the setting of probable infection, the situation is more complex with treatment consisting probably of implant exchange and prolonged antimicrobial treatment. Nevertheless, several authors reported good outcomes with simple surveillance following “aseptic” implant replacement in cases of probable *C. acnes* infection. Specifically, Dramis et al. [34] in a retrospective study of 50 patients with a single positive *C. acnes* culture during revision TJR due to pain and/or loosening, 15 treated with prolonged postoperative antibiotics and 35 with simple surveillance, reported a single revision surgery due to infection relapse, at an average 20.5-month follow-up, in a patient who received 6-week postoperative antibiotics. Similarly, Grosso et al. [35] reported a 5.9% (1/17) infection relapse in patients treated with one-stage “aseptic” TJR revision and a *C. acnes*-positive intraoperative culture, treated without prolonged antibiotics. In contrast, Tsukayama et al. [36], who systematically provided prolonged antimicrobial therapy in all patients with positive intraoperative samples, reported poor results, with 16% (5/31) relapse rate. In the present study, 24 patients demonstrated a single positive intraoperative culture, with 8/24 (33.3%) classified as PJI based on the presence of local signs of infection ± increased inflammatory markers. All the patients with a single positive culture, who were classified as PJI, received a prolonged antibiotic treatment, whereas 62.5% (10/16) of the patients who were classified as contamination received a prolonged antibiotic treatment, with no infection relapse in either group, at a minimum 2-year follow-up.

Preoperative TJR aspiration might demonstrate a low sensitivity but a high specificity in detecting *C. acnes* PJI. Frangiamore et al. [37] in a retrospective study of 202 consecutive patients with painful shoulder arthroplasty reported a 26.3% sensitivity and 87.5% specificity of preoperative TJR aspiration in detecting *C. acnes* PJI confirmed by positive intraoperative cultures. Similarly, Hecker et al. [38] reported that the sensitivity, specificity, PPV and NPV of preoperative aspirations in 106 shoulder aspirations (native, post-fracture repair, post-arthroscopy and post-arthroplasty) with saline irrigation was 33%, 98%, 80% and 83%, respectively. The present study reported a 59% sensitivity and 88% specificity for preoperative TJR aspiration in detecting *C. acnes* PJI. In PJIs with other pathogens, a preoperative TJR aspiration might, however, demonstrate a higher diagnostic accuracy compared to *C. acnes* PJI. Qu et al. [39] in a metanalysis of 34 studies including 3332 patients reported a 70% sensitivity and 94% specificity for THA, whereas a 78% sensitivity and 96% specificity for TKA, respectively. Similarly, Fink et al. [40] in a prospective study of 145 TKA, who underwent revision surgery for component loosening, reported a 72.5% sensitivity, which was similar to C-reactive protein > 13.5 mg/l, and 95.2% specificity of preoperative TJR aspiration. The reportedly low sensitivity of TJR aspiration for *C. acnes* PJI could be attributed to the fact that joint aspirations might only detect planktonic bacteria and therefore yield negative results in the presence of a biofilm-forming pathogen such as *C. acnes* [13, 41]. These data might suggest that a negative TJR aspiration should not rule out a *C. acnes* PJI, especially in the presence of clinical correlates of infection.

The current study should be interpreted in light of its potential limitations. The main drawback was the retrospective design. To obtain a sufficient number of cases, a long observation period was performed, which might have resulted in a heterogenous diagnostic workup and treatment concepts. Nevertheless, the relevant parameters were documented concisely and uniformly. Finally, the sample size number of the collective was still relatively small to reliably report on the discussed problems. However, it should be noted that *C. acnes* PJI are scarce and the present study is one of the largest cohorts to report on the treatment outcomes of *C. acnes* contamination or infection in the presence of a TJR.

## Conclusion

The present study reported the treatment outcomes of patients with *C. acnes* contamination or infection in the presence of a TJR. At a minimum follow-up of 24 months, infection eradication of *C. acnes* PJI was 97%, suggesting that these patients could be adequately treated with a combination of revision surgery and prolonged postoperative antibiotics. The diagnosis of *C. acnes* PJI might be challenging with more than one-quarter of patients presenting without suspicion of *C. acnes* PJI. The appropriate treatment of patients with a single positive culture remains still unclear. Nevertheless, the infection relapse rate in these is low regardless of the treatment regimen (prolonged antibiotic or simple surveillance). A negative TJR aspiration should not rule out a *C. acnes* PJI, especially in the presence of clinical correlates of infection.

## Data Availability

The materials described in the manuscript, including all relevant raw data, will be freely available to any researcher wishing to use them for non-commercial purposes, without breaching participant confidentiality.
